# The risk of vancomycin toxicity in patients with liver impairment

**DOI:** 10.1186/s12941-020-00354-2

**Published:** 2020-03-31

**Authors:** Luigi Brunetti, Jong Hwa Song, David Suh, Heui Jae Kim, Yeon Hee Seong, Dae Song Lee, Seung-Mi Lee, Dong-Churl Suh

**Affiliations:** 1 Department of Pharmacy Practice and Administration, Ernest Mario School of Pharmacy, Piscataway, NJ USA; 2 Department of Pharmacy, RWJ Barnabas Health-Robert Wood Johnson University Hospital Somerset, Somerville, NJ USA; 3 Department of Pharmacy Administration, Chung-Ang University College of Pharmacy, Seoul, South Korea; 4 School of Public Health, University of Michigan, Ann Arbor, MI USA; 5 Department of Pharmacy, College of Pharmacy, Daegu Catholic University, Gyeongsan-si, Gyeongsangbuk-do South Korea

**Keywords:** Vancomycin, Toxicity, Liver dysfunction, Kidney injury

## Abstract

**Background:**

The influence of liver disease on the pharmacokinetic profile, the risk of acute kidney injury, and excessive drug exposure in patients treated with vancomycin was examined.

**Methods:**

A retrospective cohort study was performed with patients discharged from a medical center between January 2011 and June 2018 who received vancomycin therapy. Patients were stratified according to liver dysfunction (no to mild liver dysfunction (NMLD) and moderate to severe liver dysfunction (MSLD) based on the Child–Pugh score. The risk of acute kidney injury was compared between patients who were stratified by the attainment of a target serum trough concentration (10 mg/dL to 20 mg/dL) and the vancomycin ratio formed between the area under the curve and minimum inhibitory concentration. The impact of liver dysfunction and a daily dose of vancomycin on the risk of acute kidney injury and vancomycin AUC:MIC > 600 were tested using logistic regression with and without adjusting for the study variables.

**Results:**

A total of 408 patients empirically treated with vancomycin were included in this study (237 with NMLD and 171 with MSLD). Mean vancomycin trough concentrations (17.5 ± 8.4 mg/dL versus 15.3 ± 5.2 mg/dL, p = 0.0049) and AUC:MIC ratios (549.4 ± 217.2 versus 497.5 ± 117.3, 0.0065) were significantly higher in the MSLD group when compared to the NMLD group, respectively. Vancomycin clearance was also lower in the MSLD group and corresponded to a longer half-life. The proportion of patients who developed acute kidney injury was greater in patients with MSLD when compared to NMLD (7.6% versus 3.8%, respectively; p = 0.0932); however, the difference was statistically insignificant. Furthermore, supratherapeutic serum trough concentrations and AUC:MIC ratios were more common in the MSLD group versus the NMLD group (27.5% versus 13.9%, p = 0.0007 and 28.7% versus 17.3%, respectively; p = 0.0063).

**Conclusions:**

MSLD correlates with an increased risk of supratherapeutic vancomycin exposure. Although patients with MSLD had a higher risk of acute kidney injury, the difference was not significant.

## Background

Vancomycin (a glycopeptide antibiotic) has been used to treat serious gram-positive infections, specifically methicillin-resistant *Staphylococcus aureus* [1, 2]. Earlier formulations of vancomycin were associated with significant toxicity, particularly renal toxicity, which was later discovered to be related to product impurities [1, 3]. Vancomycin is frequently used in healthcare settings, and, despite the development of refined formulations, reports of nephrotoxicity with incidence rates ranging from 5 to 43% have been reported [4–6].

A direct relationship exists between the vancomycin trough concentration and nephrotoxicity, with a greater risk occurring in patients with trough concentrations greater than 15 μg/mL [6]. Supratherapeutic vancomycin trough concentrations are associated with increased rates of nephrotoxicity, ototoxicity, and mortality [7, 8]. Current evidence supports the use of the area under the curve: minimum inhibitory concentration (AUC:MIC) ratio to monitor vancomycin therapy with a target ratio of 400 to 600 [9–11]. An AUC:MIC greater than 600 is associated with an increased risk of acute kidney injury [12]. Serum vancomycin trough concentrations are used as a surrogate to achieve an AUC:MIC ratio of ≥ 400, the pharmacodynamic parameter that has been identified as the primary predictor of vancomycin effectiveness [1, 2]. Moise-Broder and colleagues were among the first to associate vancomycin efficacy with maintaining an AUC/MIC ≥ 400.

Drug clearance is an important factor in determining AUC. Estimating clearance in patients with liver disease is often challenging because the standard approach to estimating renal function (Cockcroft-Gault) relies on serum creatinine. Because serum creatinine is dependent on muscle mass, and individuals with liver disease often have low muscle mass, renal function is often over-estimated [13]. Ultimately, optimal vancomycin dosing and concurrent monitoring of renal function are paramount for decreasing the occurrence of toxicities [2].

Renal function is often acknowledged when selecting a vancomycin dosing strategy; however, liver function is not commonly considered. Furthermore, data to support the influence of liver dysfunction on vancomycin pharmacokinetics are limited [14–16]. One study reported alterations in vancomycin half-life and clearance (i.e. showing a much longer half-life and decreased clearance in patients with impaired hepatic function when compared to those with normal hepatic function) [14]. In addition, subjects with significant hypoalbuminemia (commonly present in severe liver disease) display a prolonged vancomycin half-life and a greater incidence of nephrotoxicity [17]. The objectives of this study were to evaluate the distribution of the vancomycin key pharmacokinetic parameters in patients treated with vancomycin, to investigate the influence of liver dysfunction (i.e. moderate to severe versus no to mild liver disease) on the risk of nephrotoxicity during treatment with vancomycin, and to identify factors influencing acute kidney injury and vancomycin trough concentration.

## Materials and methods

### Study design and data collection

A retrospective cohort study was performed using patient records gathered from a pharmacy-managed vancomycin pharmacokinetic service in an academic community medical center with 365 beds located in Somerville, New Jersey, USA between January 2011 and June 2018. Patients who were treated with vancomycin during hospitalization were screened.

Data extracted included age; gender; race; creatinine clearance (CrCl); body mass index (BMI); vancomycin dosing strength and frequency; vancomycin through level; estimates of liver function; comorbidities identified using International Classification of Diseases codes; procedures; length of hospitalization; mortality; Child–Pugh scores; and relevant laboratory values (e.g., albumin). Child–Pugh scores were used to stratify patients into groups based on liver dysfunction: no to mild liver dysfunction (NMLD, Child–Pugh Class A, 5 6) or moderate to severe liver dysfunction (MSLD, Child–Pugh Class B or C, 7 15) [18]. This protocol for this study was reviewed and approved by the Robert Wood Johnson Somerset Institutional Review Board (IRB Protocol number IRB18-08).

### Patient inclusion and exclusion criteria

Assuming a 10% rate of supratherapeutic vancomycin trough concentrations, a total of 42 patients in each group would provide a power of 90% in detecting a relative difference in the proportion of patients reaching supratherapeutic concentrations of 30% with a two-sided alpha level of 0.05 [19]. Patients who were 18 years or older and received vancomycin treatment during the hospital stay were eligible for study inclusion. Patients with a CrCl greater than 40 mL/min (calculated using the Cockcroft-Gault equation) at baseline and a vancomycin trough concentration drawn prior to at least the third dose were included in the study because only these patients were managed using the protocol at the study site. If patients were readmitted, only their first admission was included. Patients receiving one-time doses, hemodialysis, or surgical prophylaxis were excluded from the study. Pregnant patients were also excluded.

A total of 414 eligible patients receiving vancomycin therapy were screened for inclusion into the study; 6 were excluded because they were missing variables needed to calculate liver dysfunction severity. This study included a final total of 408 patients.

### Study outcomes

The primary outcome of this study was the incidence of patients with acute kidney injury while on vancomycin therapy. Acute kidney injury was defined as a ≥ 0.5 mg/dL rise in serum creatinine or a 50% increase above baseline (whichever was greater) through the course of therapy for two or more consecutive days [2]. Secondary endpoints included serum trough concentration, AUC:MIC at steady-state, and supratherapeutic concentrations (defined as trough concentration > 20 μg/mL or AUC:MIC > 600) [2, 20, 21].

Trough concentrations were drawn thirty minutes prior to the upcoming dose, with a ±  1-hour leeway. If the trough concentration was drawn outside the 1-hour window, the trough was extrapolated to 30 min prior to the next scheduled dose using standard pharmacokinetic calculations with an estimated elimination rate constant and half-life. The area under the curve was estimated with DoseMe software, which uses a Bayesian approach, as previously described [22]. The minimum inhibitory concentration was conservatively estimated to be 1 in order to calculate the AUC:MIC ratio [23].

### Statistical analysis

The differences in the baseline characteristics, vancomycin dosing variables, and clinical outcomes stratified by the level of liver dysfunction (NMLD group vs. MSLD group) were tested using the *t* test for continuous data and the Chi square test or Fishers’ exact test for categorical data. One-way analysis of variance was used to test the differences in the means of outcome values among the three groups stratified by the level of vancomycin as noted in the AUC:MIC ratio.

Normality testing was performed using the Shapiro–Wilk test. If data were not normally distributed, the Mann–Whitney U test or Kruskal–Wallis Rank Sum test was used to compare the medians. Pearson correlation coefficients were calculated to test possible correlations between vancomycin trough concentration and key pharmacokinetic parameters including vancomycin ratio of AUC:MIC, clearance (ml/min), the volume of distribution per weight (L/kg), and half-life (hour).

Logistic regression analysis was performed to determine factors which influence the occurrence of acute kidney injury, supratherapeutic vancomycin concentration, and mortality, vancomycin AUC:MIC ratio of > 600, with and without adjusting for the severity of liver dysfunction, daily dose of vancomycin used, sex, age, body mass index(kg/m [2]), Charlson comorbidity index score, and comorbidities including cardiovascular diseases and cancer. The Charlson comorbidity index was calculated using ICD-9 codes as a proxy for patients’ comorbid disease burden [24]. Confounders included variables that were established in the literature as clinically meaningful. Data analyses were performed using SAS 9.4 (SAS Institute Inc., Cary, NC).

## Results

Of the 408 patients included in the study, 237 had NMLD and 171 had MSLD. Patient demographics and dosing characteristics are summarized by the level of liver dysfunction in Table 1. The groups had a different mean age (55.4 ± 17.1 years for NMLD, versus 59.3 ± 15.3 years for MSLD, p = 0.0169). Mean CrCl-based estimates of kidney function were significantly higher in the MSLD group, but the mean baseline CrCl was similar between the groups. A higher percentage of patients had lower CrCl (30 60 mL/min) in the MSLD group, and serum albumin was also significantly lower in the MSLD group. The occurrence of acute kidney injury was higher in patients with MSLD when compared to those with NMLD (7.6% versus 3.8%, respectively; p = 0.0932), but the difference was statistically insignificant. Mortality (14.0% versus 4.2%; p = 0.0004) and length of hospital stay (13.9 days versus 8.6 days; p < 0.0001) were significantly higher in patients with MSLD when compared to those with NMLD, respectively (Table 2). The proportion of patients with a supratherapeutic trough concentration (> 20 µg/mL) was significantly greater in the MSLD group when compared to the NMLD group (27.5% versus 13.9%, respectively; p = 0.0007). The initial mean trough concentrations were higher in the MSLD group versus the NMLD group. The proportion of patients with a supratherapeutic AUC:MIC (> 600) was also significantly higher in the MSLD group when compared to the NMLD group (28.7% versus 17.3%, respectively).

**Table 1:** Baseline characteristics and dosing variables by level of liver dysfunction

Variable	No to mild liver dysfunction (n = 237) n (%)	Moderate to severe liver dysfunction (n = 171) n (%)	P-value
Age (years)			
Mean ± SD	55.4 ± 17.1	59.3 ± 15.3	0.0169
18 –44	61 (25.7)	26 (15.2)	0.0354
45 –64	104 (43.9)	83 (48.5)	
≥ 65	72 (30.4)	62 (36.3)	
Female (%)	74 (31.2)	59 (34.5)	0.4856
Body mass index (kg/m^2^)			
Mean ± SD	30.5 ± 9.5	29.6 ± 9.9	0.0532
< 25	62 (26.2)	64 (37.4)	0.0507
25–29.9	71 (30.2)	45 (26.3)	
≥ 30	104 (43.9)	62 (36.3)	
Vancomycin total daily dose (mg)			
Mean ± SD	3030 ± 969	2768 ± 1014	0.0082
750–2000	62 (26.2)	63 (36.8)	0.0412
2001–3000	86 (36.3)	61 (35.7)	
3001–4000	50 (21.1)	21 (12.3)	
> 4000	39 (16.5)	26 (15.2)	
Vancomycin dosing frequency			
Every 8 h	140 (59.1)	80 (46.8)	0.0363
Every 12 h	95 (40.1)	88 (51.5)	
Every 24 h	2 (0.8)	3 (1.8)	
Charlson comorbidity index, mean ± SD	1.7 ± 2.1	2.7 ± 2.5	< 0.0001
Comorbidities			
Cardiovascular diseases	67 (28.3)	61 (35.7)	0.1118
Cancer	17 (7.2)	36 (21.1)	< 0.0001
Anemia	96 (40.5)	134 (78.4)	< 0.0001
Creatinine clearance (mL/min)			
Mean ± SD	82.8 ± 29.8	79.1 ± 34.9	0.0272
30 –59	48 (20.3)	60 (35.1)	0.0032
60– 89	108 (45.6)	60 (35.1)	
≥ 90	81 (34.2)	51 (29.8)	
Albumin (g/dL)	3.5 ± 0.5	2.3 ± 0.6	< 0.0001

*SD* standard deviation

**Table 2:** Clinical outcomes by level of liver dysfunction

Variable	No to mild liver dysfunction (n = 237) n (%)	Moderate to severe liver dysfunction (n = 171) n (%)	P-value
Acute kidney injury	9 (3.8)	13 (7.6)	0.0932
Mortality	10 (4.2)	24 (14.0)	0.0004
Length of hospital stay, mean ± SD (days)	8.6 ± 7.3	13.9 ± 10.6	< 0.0001
Vancomycin trough concentration (µg/mL)			
Mean ± SD	15.3 ± 5.2	17.5 ± 8.4	0.0049
Trough < 10	30 (12.7)	19 (11.1)	0.0024
10 ≤ Trough ≤ 20	174 (73.4)	105 (61.4)	
Trough > 20	33 (13.9)	47 (27.5)	
Vancomycin AUC:MIC ratio			
Mean ± SD	497.5 ± 117.3	549.4 ± 217.2	0.0065
AUC:MIC < 400	40 (16.9)	31 (18.1)	0.0142
400 ≤ AUC:MIC ≤ 600	156 (65.8)	91 (53.2)	
AUC:MIC > 600	41 (17.3)	49 (28.7)	
Vancomycin volume of distribution (L)	82.6 ± 25.2	79.8 ± 24.5	0.2120
Vancomycin volume of distribution per weight (L/kg)	0.94 ± 0.13	0.93 ± 0.12	0.3622
Vancomycin clearance (ml/min)	5.8 ± 2.4	4.8 ± 2.0	< 0.0001
Vancomycin half-life (hour)	11.3 ± 5.1	13.4 ± 6.5	0.0012
Vancomycin K-elimination (ml/min)	0.074 ± 0.035	0.063 ± 0.027	0.0012

*SD* standard deviation, *AUC*: area under curve for 24 h, *MIC* minimum inhibitory concentration

**Fig. 1:**
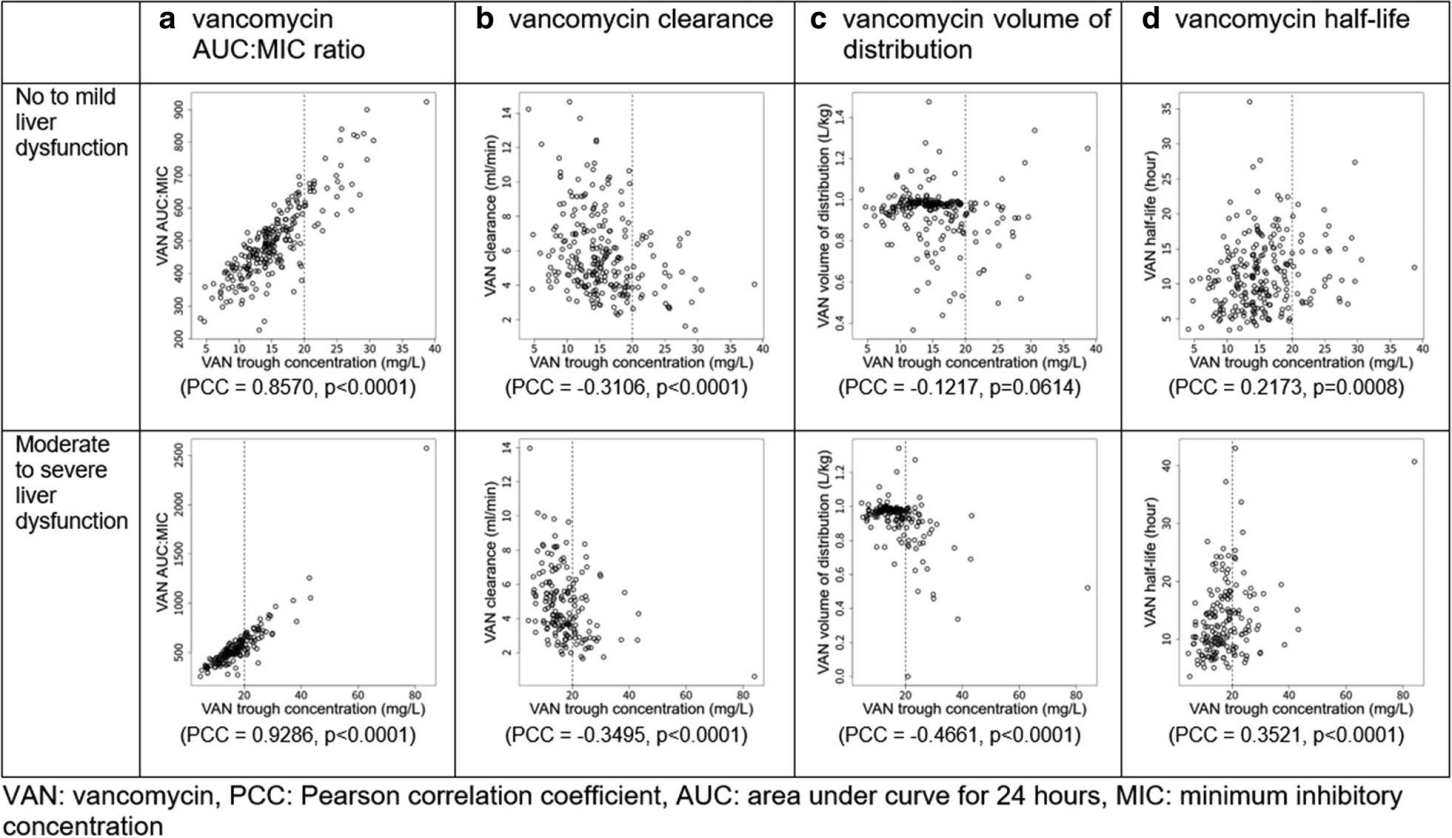
Correlation between vancomycin trough concentration and four pharmacokinetic parameters

Patients’ vancomycin trough concentrations and clearance increased linearly as their AUC:MIC ratios increased (Table 3). Vancomycin trough concentration positively correlated with AUC:MIC and half-life (correlation coefficients = 0.9059 and 0.3162, respectively; all p < 0.0001) and negatively correlated with clearance and volume of distrib½ution per weight (correlation coefficients = − 0.3368 and − 0.3151, respectively; all p < 0.0001) (Fig. 1).

**Table 3:** Patient outcomes by area under the curve for 24 h

Variable	AUC:MIC < 400	400 ≤ AUC:MIC ≤ 600	AUC:MIC > 600	P-value
	(n = 71)	(n = 247)	(n = 90)	
Acute kidney injury, n (%)	3 (4.2)	11 (4.5)	8 (8.9)	0.2879
Mortality, n (%)	10 (14.1)	17 (6.9)	7 (7.8)	0.1502
Length of hospital stay, mean ± SD (days)	11.5 ± 7.6	9.8 ± 9.1	13.0 ± 10.5	0.0015
Vancomycin trough concentration, mean ± SD (µg/mL)	10.4 ± 3.8	14.9 ± 3.3	24.5 ± 8.4	< 0.0001
Vancomycin clearance (ml/min)	6.6 ± 3.2	5.5 ± 2.0	4.1 ± 1.5	< 0.0001

P-value was calculated using Fisher’s exact test for categorical data and Kruskal–Wallis rank sum test for continuous data

*SD* standard deviation, *AUC* area under the curve for 24 h, *MIC* minimum inhibitory concentration

After adjusting for sex, age, and total daily dose of vancomycin, body mass index, and Charlson comorbidity index score, the incidence of acute kidney injury was 2.48 times (95% CI: 0.98–6.28) higher in patients with MSLD than those with NMLD, but the impact was statistically insignificant (Table 4). However, the severity of patient’s liver dysfunction significantly influenced the likelihood of a vancomycin supratherapeutic trough concentration (adjusted odds ratio = 2.31, 95% CI 1.36–3.93) and vancomycin supratherapeutic ratio of AUC:MIC (adjusted odds ratio = 1.79, 95% CI 1.09 2.96) after adjusting for the study variables.

**Table 4:** Likelihood of clinical outcomes by liver dysfunction in patients with vancomycin (adjusted odds ratios and their 95% confidence intervals)

Variable	Acute kidney injury		Mortality		Vancomycin trough concentration > 20 µg/mL		Vancomycin AUC:MIC > 600	
Liver dysfunction								
No to mild	1		1		1		1	
Moderate to severe	2.48	(0.98- 6.28)	2.79	(1.26-6.18)	2.31	(1.36 -3.93)	1.79	(1.09-2.96)
Daily dose of vancomycin								
< 3000 mg	1		1		1		1	
≥ 3000 mg	1.85	(0.68-5.07)	0.46	(0.19-1.09)	2.76	(1.51-5.04)	1.59	(0.91-2.77)
Gender								
Female	1		1		1		1	
Male	4.99	(1.13-22.05)	1.36	(0.59-3.14)	0.69	(0.40-1.19)	0.58	(0.35-0.96)
Age (years)								
18–64	1		1		1		1	
≥ 65	1.99	(0.74-5.40)	1.48	(0.66-3.33)	1.43	(0.78-2.63)	0.91	(0.50-1.65)
Body mass index (kg/m^2^)								
< 25	1		1		1		1	
25–29.9	0.62	(0.19-2.04)	1.22	(0.48-3.06)	0.46	(0.22-0.93)	0.65	(0.35-1.18)
≥ 30	0.95	(0.34-2.61)	0.76	(0.32-1.83)	0.72	(0.40-1.29)	0.37	(0.21-0.68)
Charlson comorbidity index								
0-1	1		1		1		1	
≥ 2	0.73	(0.29 -1.83)	2.72	(1.15-6.42)	1.68	(0.98-2.87)	1.26	(0.76-2.09)

*AUC* area under curve for 24 h, *MIC* minimum inhibitory concentration

## Discussion

This study is one of the few cohort studies that evaluates the use of vancomycin in patients with liver dysfunction. While pharmacokinetic parameters have been studied previously, the influence of pharmacokinetic alterations on the attainment of therapeutic vancomycin concentrations has not been extensively studied in this patient population.

The proportion of patients with supratherapeutic vancomycin exposure was more than double in patients with MSLD versus patients with NMLD. Vancomycin clearance was reduced in patients with MSLD when compared to patients with NMLD, and this corresponded to an increased half-life. Both of these pharmacokinetic parameters may influence overall drug exposure. One important consideration is the estimation of renal function in patients with liver disease. A key variable in the Cockcroft-Gault equation is serum creatinine. Patients with liver disease often suffer from malnutrition and decreased muscle mass; therefore, they may have low serum creatinine, resulting in an overestimation of renal function. This is evident in our analysis where patients in the MSLD group had significantly lower serum albumin, possibly suggesting reduced muscle mass. This phenomenon may have led to an overestimation of renal function and the subsequent increase in the prevalence of supratherapeutic trough concentrations we observed in the MSLD group. In addition, hypoalbuminemia is associated with a prolonged vancomycin half-life [17]. Based on the aforementioned postulation and data, a reduction in frequency seems to be a reasonable approach when dosing vancomycin in patients with significant liver disease. Of note, decreased albumin concentration may also be the result of decreased protein production due to hepatic dysfunction and hypoalbuminemia caused by critical illness. Future studies are needed to support this hypothesis.

Patients classified with more advanced liver disease (i.e. MSLD, Child–Pugh Class B or C) had an increased mean vancomycin trough concentration as well as a higher prevalence of a supratherapeutic trough concentration when compared to those with no or less severe liver disease (i.e. NMLD). Previous studies have shown a correlation between increased trough concentration and the incidence of adverse drug reactions, particularly nephrotoxicity [4, 25].

Acute kidney injury is a well-known, adverse drug reaction that may occur secondary to vancomycin exposure. The development of acute kidney injury includes a variety of risk factors as reported in published literature, including concomitant nephrotoxins, advanced age, total daily doses exceeding 4 grams, and hypotension [26, 27]. Traditionally, serum trough concentration exceeding 15 mg/dL has been thought of as a risk for acute kidney injury, but recent studies have provided guidance on AUC/MIC thresholds for minimizing nephrotoxicity [28]. Chavada and colleagues reported that an AUC:MIC ratio > 563 was associated with a significant increase in nephrotoxicity (40% versus 11.2%; p = 0.002) [29]. Similarly, Zasowski and colleagues reported a 3-to-4-fold increase in nephrotoxicity when the AUC:MIC ratio exceeded 700, even after controlling for other risk factors [30]. In comparison, our study suggests that individuals with liver disease are likely to exceed these AUC:MIC ratio thresholds.

A vancomycin ratio of AUC:MIC > 600 occurred more often in MSLD patients than NMLD patients. Regardless of the metric being used (trough or AUC:MIC), the prevalence of a supratherapeutic concentration in this patient population is of concern. Unlike previous studies that have evaluated risk factors for toxicity, this present study evaluates both liver disease as a chronic condition and drug exposures as defined by serum trough concentration and a/the AUC:MIC ratio. The secondary clinical outcomes (e.g. mortality and length of stay) were significantly higher in the more severely ill patients. Acute kidney injury was more common in the MSLD group versus the NMLD group. The difference was not significant and was likely related to a type II error given the wide confidence interval and the low overall incidence of acute kidney injury in the studied cohort (5.4%). Another concern regarding vancomycin therapy and supratherapeutic trough concentration is the incidence of ototoxicity [8, 31]. Due to the retrospective nature of this study, this adverse drug reaction was not observed in this specific patient cohort.

The higher rate of acute kidney injury, mortality, and length of stay in the MSLD group may not be attributed solely to vancomycin therapy and subsequent trough concentration. The severity of illness (particularly liver disease) also influences the length of stay, acute kidney injury, and mortality rates. Furthermore, patients with liver dysfunction can have a concomitant occurrence of hepatorenal syndrome. Hepatorenal syndrome is a reversible renal impairment that can occur in those with advanced liver cirrhosis or fulminant hepatic failure [32]. Although the occurrence of this pathology was not studied in this cohort, hepatorenal syndrome can influence the risk of nephrotoxicity when vancomycin is administered. Of note, only patients with a CrCl > 40 mL/min at baseline were included in this analysis, and baseline renal function was similar between the two groups. In addition, only eight patients in the study had a Child–Pugh score of greater than 9 (Class C). The use of other nephrotoxic agents along with vancomycin can also have an added negative effect on renal function, and the current study did not take these medications into consideration. Further studies should account for these variables when clinically assessing the outcomes of vancomycin usage in patients with liver dysfunction.

There are some limitations to the study that should be considered. The influence of concomitant medications, particularly nephrotoxins, were not included in the analysis due to the lack of data availability. Diuretics are known to increase risk of acute kidney injury and are recommended by clinical practice guidelines for the management of ascites in advanced liver disease [33, 34]. Their use is essential in patients with advanced liver disease. Nonetheless, the acknowledgement that diuretic use and other risk factors are often present in individuals with liver disease further strengthens the need to exercise prudence when managing vancomycin therapy in this patient population. To control for confounding, outcomes were analyzed after adjusting for the study variables. By virtue of similar treatments undergone among patients with the same disease, the strategy used in this study may have also accounted for medication use; however, one cannot definitively exclude the possibility that concomitant therapies may have confounded the results. It is also important to control for comorbidities such as diabetes and heart failure since they can alter kidney function. The Charlson comorbidity index, which was included in the analysis, includes both diabetes and heart failure. As with any retrospective cohort study, the potential for information bias associated with the extracted medical information from the electronic health records exists. To minimize the risk of information bias, data were extracted electronically and then confirmed through manual review of the electronic health records.

## Conclusion

This study examined the influence of pharmacokinetic parameters in patients with MSLD. While vancomycin half-life was prolonged, clearance decreased in this patient population, which led to an increase in supratherapeutic exposure. Although MSLD was associated with an increased risk of nephrotoxicity, the increased risk compared to individuals with NMLD was not statistically significant. When initiating vancomycin treatment in patients with liver dysfunction, clinicians should consider dosage and/or frequency reductions to minimize the occurrence of supratherapeutic concentrations and potential adverse consequences. Future studies are needed to determine if dosage adjustments improve patient outcomes and if patients with liver disease are at greater risk of vancomycin related toxicity.

## Data Availability

The datasets used and/or analyzed during the current study are available from the corresponding author upon request.
